# Resolution of preeclampsia after selective termination in discordant twins: A case report and literature review

**DOI:** 10.1097/MD.0000000000031484

**Published:** 2022-11-25

**Authors:** Hua Liao, Zhaomin Zeng, Hongyan Liu, Qing Hu, Haiyan Yu

**Affiliations:** a Department of Obstetrics and Gynecology, West China Second University Hospital, Sichuan University, Chengdu, China; b Key Laboratory of Birth Defects and Related Diseases of Women and Children (Sichuan University), Ministry of Education, Chengdu, China.

**Keywords:** discordant twins, literature review, preeclampsia, selective termination, twin pregnancy

## Abstract

Preeclampsia is a major cause of maternal and perinatal morbidity and mortality. Growth discordance is associated with an increased risk of preeclampsia in twin pregnancies. The management of preeclampsia combined with discordant twins is difficult and controversial because of severe maternal conditions and severe growth restriction in 1 twin.

**Patient concerns:**

A 34-year-old woman with dichorionic diamniotic twin pregnancy complicated by preeclampsia at 29 weeks of gestation, and 1 twin with severe growth restriction and fetal intracranial hemorrhage.

**Diagnosis:**

The patient developed severe preeclampsia with high blood pressure (>160/100 mm Hg) and proteinuria, hydrothorax and leg edema. Ultrasound examination confirmed growth restriction (weight estimation: 915 g, <1st percentile) and abnormal umbilical flow in 1 twin (twin B), with a normal co-twin (estimated weight: 1693 g) (twin A). Magnetic resonance imaging revealed intracranial hemorrhage in the germinal matrix of twin B.

**Interventions:**

Selective termination of twin B by intracardiac injection of potassium chloride was performed at 31 weeks and 2 days’ gestation.

**Outcomes:**

Symptoms of preeclampsia resolved after selective termination, allowing the pregnancy to be prolonged for nearly 4 weeks. A healthy female infant was delivered at 35 weeks of gestation.

**Conclusion:**

Delivery of both fetus is not the only choice for the management for twin pregnancy with severe preeclampsia and discordant twins. Selective termination of the fetus with poor prognosis could be a reasonable treatment choice in carefully selected cases.

## 1. Introduction

Preeclampsia is one of the hypertensive disorders of pregnancy which is the leading causes of maternal and perinatal mortality worldwide. The prevalence of hypertensive disorders of pregnancy is around 2% to 8% of all pregnancies.^[1]^ Twin pregnancies have a three-to-four-fold increased risk of preeclampsia compared to singleton pregnancies,^[2]^ with an overall rate of around 9.5%. The exact etiology and pathogenesis of preeclampsia is not completely understood and thereby the ultimately effective methods for the prevention and treatment remains to terminate the pregnancy.

Currently, the relationship between preeclampsia and growth discordance in twin pregnancies has been investigated. Studies have revealed that growth discordance in twins is associated with an increased risk for preeclampsia.^[3–5]^ Therefore, the clinical management of such cases is complex, especially in patients with serious maternal condition due to preeclampsia and 1 twin in severe growth restriction. Traditionally, delivery of the both fetus and placenta is the only known effective cure in these cases. It is a great challenge for staff to prolong the pregnancy for improve the outcome. So far, there are only a few published articles relevant to preeclampsia resolving after selective termination in discordant multiple gestation and they are case reports.

Herein, we reported a rare case of preeclampsia resolution after selective termination of the growth-restricted twin complicated with fetal intracranial hemorrhage in a dichorionic diamniotic twin pregnancy, in which prolonged the pregnancy. The study aims to further contributes to the literature and to investigate an alternative approach which allows the survival of the normal twin. In addition, we used a list of keywords including “preeclampsia,” “twins,” “discordant twins,” “twin pregnancy,” “selective termination,” “feticide,” and “multiple pregnancy” to perform an extensive Medline search and conducted a literature review. This study was approved by the ethical committees at the West China Second University Hospital of Sichuan University.

## 2. Case report

A patient was a 34-year-old woman, gravida 2, para 0, who conceived a trichorionic triplet gestation by in vitro fertilization and embryo transfer (IVF-ET). One vanishing embryo occurred at 7 gestational weeks, which resulted in dichorionic diamniotic twin pregnancy. At 23 weeks’ gestation, fetal ultrasonography revealed a small cavum septum pellucidum in twin B, as well as inconsistent growth of the 2 fetuses with twin B lagging by 1 week. Fetal brain magnetic resonance imaging (MRI) showed a nodular shadow near the left lateral ventricle of twin B, suggesting a hemorrhage within the germinal matrix. Amniocentesis was performed and revealed no chromosomal abnormalities both twins.

The patient had no previous history or family history of hypertension, and during the first and second trimesters of her pregnancy, her blood pressure was normal, ranging between 98 to 128 and 65 to 82 mm Hg. At 29 weeks’ gestation, she presented with edema and a newly elevated blood pressure at 110 to 161 and 73 to 113 mm Hg as measured by 24-hour ambulatory blood pressure monitoring and an elevated serum total bile acid of 22.8µmol/L. The urine protein excretion was 1.34 g/24 hours. Preeclampsia was diagnosed, and magnesium sulfate was given, describing labetalol 200 mg orally 3 times a day, combined with nifedipine 30 mg orally once a day and glucocorticoid to promote fetal lung maturation.

**Figure 1.**
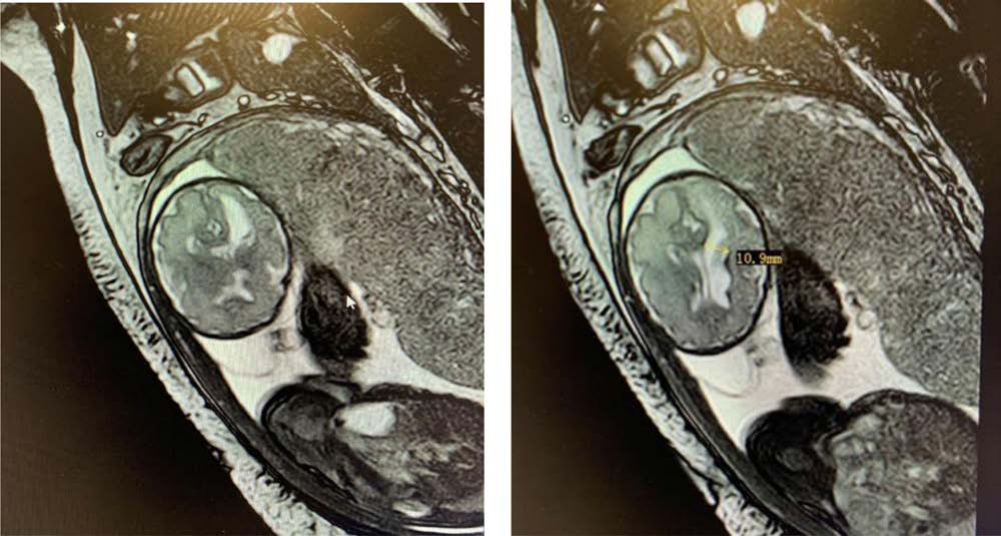
Magnetic resonance image of intracranial hemorrhagic lesions of twin B at 30 wk.

At 30 weeks’ gestation, the result of follow-up MRI revealed twin B with a small hemorrhagic focus in the left ventricle, resulting in mildly dilated left ventricle, dorsal thalamus, and cerebral peduncle volume reductions (Fig. 1). At 31 weeks’ gestation, fetal ultrasonography showed that twin A was growing normally with estimated fetal weight (EFW) 1693 g while twin B was lagging by 5 weeks with EFW 915g (<1 percentile). The umbilical artery flow in twin A was PI 1.06 and S/D 2.96, in twin B PI 1.33 and S/D 4.0 to 4.18. She presented with hydrothorax and edema. The patient’s liver function was abnormal with an elevated total serum bile acid of 16.8 umol/L. The uric acid was 544 umol/L, and urinary protein excretion increased as 5.693 g/24 hours. She was hemoconcentrated with hematocrit 41%.

**Figure 2.**
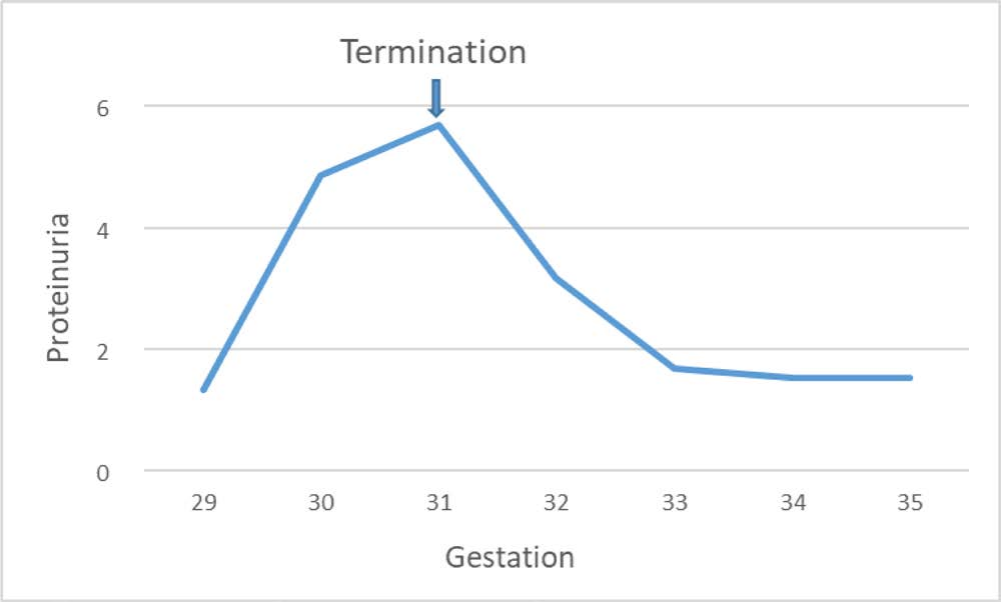
Degree of proteinuria before and after selective termination.

The couple were extensively counseled by multidisciplinary teams about the maternal condition, the twin’s status and the treatment, especially the poor prognosis of twin B. The couple opted selective termination of twin B to prolong the pregnancy. Selective fetal reduction by intracardiac injection of potassium chloride was performed to twin B at 31 weeks and 2 days’ gestation. After fetal reduction, the patient’s symptoms of preeclampsia gradually resolved, meanwhile, the urine protein quantification decreased gradually (Fig. 2), and the total serum bile acid level and liver function and uric acid normalized. One week after selective termination, her blood pressure remained normal with a low dose of oral nicardipine (30 mg per day). Pulsatility index and resistance index in umbilical cord blood flow monitoring remained normal in the surviving fetus.

At 35 weeks of gestation, cesarean section was performed duo to early onset of labor. A healthy female baby weighed 2200 g with Apgar scores of 10 and 10 at 1 and 5 minutes, respectively, whereas the dead fetus weighed 1050 g. The postpartum course was uneventful and was discharged 3 days later. So far, the woman and the infant are followed up closely. The woman is in good condition. The baby is now 10 months old and is in good health.

## 3. Discussion

Preeclampsia is the leading causes of maternal and perinatal mortality worldwide. Growth discordance in twins is associated with an increased risk for preeclampsia. The clinical management of such cases is complex, especially in patients with severe or early-onset preeclampsia and a twin with severe growth restriction. The underlying pathogenesis of preeclampsia remains unclear. It is demonstrated that pregnancies with preeclampsia is associated with abnormal placentation, characterized by inefficient invasion and remodeling of the placenta spiral arteries. The ultimate cure for preeclampsia remains delivery of the fetus and placenta. It is a great challenge for staff to prolong the pregnancy for improve the prenatal outcomes.

This article reports a case of preeclampsia resolution after selective termination in a discordant twin with intracranial hemorrhage in one of the twin fetuses. It is suggested that termination the entire pregnancy is not the only choice in the management for twin pregnancy complicated with severe preeclampsia and discordant twins. Selective termination of the fetus with poor prognosis could be an alternative therapeutic strategy to prolong the pregnancy and improve perinatal outcomes in carefully selected cases.

We used a list of keywords including “preeclampsia,” “twins,” “discordant twins,” “twin pregnancy,” “selective termination,” “feticide,” and “multiple pregnancy” to perform an extensive Medline search and conducted a literature review.

There are few reported cases in the literature in which selective fetal termination has been considered as an alternative treatment for multiple pregnancies complicated by preeclampsia, especially when 1 twin has severe growth restrictions and low likelihood of survival. To the best of our knowledge, there have been only 7 cases published.^[6–10]^ Detailed information of these cases is shown in Table 1.

**Table 1:** Reported cases of preeclampsia resolution after selective termination in multiple gestations.

Study ID	Maternal age	GA of PE diagnosed (wk)	Fetal abnormality	GA at ST (wk)	Time to PE resolution	Delivery method	Delivery age (wk)	Outcome of the alive fetus
Audibert et al 2003^[6]^	32	28	Severe fetal growth discordance, twin B REDF	32	2 wk	VD	38	Live birth, healthy newborn
Gulersen et al 2022^[7]^	28	18 + 5	Severe fetal growth discordance, twin B triploid (69, XXY)	18 + 5	4 d	CS	37 + 3	Live birth, healthy newborn
Guerby et al 2019^[8]^	28	25	Severe fetal growth discordance, twin B REDF	25	1 wk	CS	29	Live birth, healthy newborn
Heyborne et al 2004^[9]^	46	26	Severe fetal growth discordance, twin B AEDF	26 + 3	<1 wk	VD	At term	Live birth, healthy newborn
	37	24 + 6	Severe fetal growth discordance, oligohydramnios, twin B AEDF	24 + 6	12 d	VD	34 + 4	Live birth, healthy newborn
	45	16	twin B hydrops	16	1–2 wk	CS	At term	Live birth, healthy newborn
Yu et al 2015^[10]^	24	26	Twin B IUGR, anhydramnios, AEDF	27 + 4	<1 wk	VD	29 + 5	Live birth, neonatal death due to pulmonary hemorrhage
This case	34	29	Severe fetal growth discordance, twin B ICH and abnormal UA Doppler	31 + 2	1 wk	CS	35	Live birth, healthy newborn

AEDF = absent end-diastolic flow, CS = cesarean section, GA = gestational age, ICH = intracranial hemorrhage, IUGR = intrauterine growth restriction, PE = preeclampsia, REDF = reversed end-diastolic flow, ST = selective termination, UA = umbilical artery, VD = vaginal delivery, wk = week.

Due to the use of assisted reproductive technologies, the incidence of twin pregnancies has increased by 70% in the last 30 years. In twin pregnancies, the rate of preeclampsia is 2 to 5 times higher than in singleton pregnancies, and has an earlier onset and higher severity.^[11]^ The prevalence of preeclampsia is 2% to 8% and 10% to 20% in singleton and twin pregnancies, respectively.^[12]^ Race, primiparity, advanced age, IVF-ET, chorionicity, and high pre-pregnancy body mass index have been found to be risk factors for preeclampsia in twins. During twin pregnancies, uterine pressure and placental ischemia cause activation of interstitial leukocytes and lipid peroxidation in the chorionic villi, aggravate immune damage and oxidative stress, damage vascular endothelial cells, and lead to hypertensive disorders in pregnancy, especially preeclampsia. Studies have suggested that the mechanism of preeclampsia complicating twin pregnancies is different from that of singleton pregnancies, and the increased risk of preeclampsia in twin pregnancies may not be related to inadequate placental perfusion, but mainly related to increased anti-angiogenic factors such as sFLT-1 caused by increased placental mass.^[11]^ Preeclampsia is thought to be associated with an increase in uteroplacental demands and a relative placental insufficiency resulting from the presence of >1 fetus. In the case presented, we speculate that termination of the compromised twin could help to control preeclampsia by stopping fetal blood flow to the abnormal placenta thus leading to decrease or arrest of the release of the substances such as sFLT-1 that are involved in the development of the preeclampsia.

It has been found that growth discordance is associated with an increased risk for preeclampsia in dichorionic twins. Studies have found that intertwin discordance of EFW ≥15%, ≥20%, ≥25% and growth restriction in 1 twin are associated with an increased incidence of hypertensive disorders in pregnancy.^[4]^ It is shown that a twin-based growth charts should be used rather than singleton growth charts to prevent overdiagnosis of intrauterine growth restriction in twin pregnancies^[13,14]^ The prevalence of preeclampsia increases with increasing degree of growth discordance, suggesting a dose-response relationship in dichorionic twin pregnancies.^[3]^ It has been demonstrated that prophylactic use of aspirin reduces the risk of preeclampsia in twins with high-risk factors. Daily intake of 150 mg of aspirin was more effective in reducing the risk of preeclampsia in twin patients in compared with 75 mg of aspirin daily.^[15]^

In our case, at first, the pregnant woman conceived the trichorionic triplet pregnancy by IVF-ET. At 7 gestational weeks, she had dichorionic diamniotic twin pregnancy due to vanishing 1 embryo. Vanishing twins/triplets were reported to occur in 15% to 35% of twin pregnancies and 50% of triplet pregnancies.^[16,17]^

Fetal intracranial hemorrhage (ICH) is a rare and life-threatening condition that occurs between 14 weeks of gestational age and delivery, with a prevalence of 0.5 to 1.0/1000. Fetal ICH can lead to fetal cerebral palsy, motor and cognitive impairment, epilepsy, microcephaly, and even fetal death.^[18]^ ICH is classified into 5 types based on site of lesion: intraventricular hemorrhage, subarachnoid, intraparenchymal, cerebellar, and subdural hemorrhage. The etiology of fetal ICH is complex and can occur spontaneously or be associated with maternal or fetal abnormalities. Fetal coagulation disorders, infections and intrauterine growth restriction, fetal gene mutation and maternal coagulation abnormalities, trauma, administration of antiepileptic drugs and anticoagulants are all associated with Fetal ICH.^[19]^ Ultrasound is the diagnostic method of choice for fetal ICH, and MRI is capable of localizing and grading hemorrhage and is an important complement to ultrasonography. In the present case, the severely growth restricted fetus was also combined with intracranial hemorrhage.

In this study, we described a rare case of resolution of preeclampsia after selective termination in a discordant dichorionic diamniotic twin pregnancy, in which the severely growth-restricted fetus (twin B) complicated with intracranial hemorrhage. Considering the extremely poor prognosis of twin B, the couple were extensively counseled by multidisciplinary teams about the maternal condition, the twin’s status and the treatment, selective termination of twin B was thought to be an alternative procedure to improve the perinatal outcome. After the procedure of selective fetocide at 31 + 2 weeks, the patient’s preeclampsia resolved, and the gestational week of the surviving fetus was extended by nearly 4 weeks. A healthy female baby was delivered at 35 weeks. The woman and the infant are followed up closely and both in good condition. This case might give some information of the mechanisms involved in the development of preeclampsia.

This study reviewed the literatures about selective termination in multiple pregnancies complicated by preeclampsia and presents one new case. The limitation of this study is the loss of detailed information in the literature review due to the lack of data in the published papers.

In conclusion, based on preeclampsia with the leading causes of maternal and perinatal mortality, it is the great challenge for staff to prolong the pregnancy to improve the perinatal outcomes in preeclampsia. Due to the rarity of selective termination in twin pregnancies complicated by preeclampsia, experience with the management is limited. The couple should be extensively counseled by multidisciplinary team about the maternal condition, the fetuses’ status and the treatment, especially in discordant twin pregnancy. Pregnancy termination is not the only choice in twin pregnancy with severe preeclampsia and discordant twins. Selective termination of the fetus with poor prognosis could be an alternative procedure in twin pregnancy to prolong the pregnancy and improve perinatal outcomes of the woman and another twin, which possibly by interrupting blood flow to the affected placenta and reducing the release of disease-causing substances. Further studies are needed for the pathophysiological mechanisms that selective termination and resolution of preeclampsia in twin pregnancy.

## Acknowledgements

We feel grateful for the doctors and staff who have been involved in this work.

## Author contributions

**Conceptualization:** Hua Liao, Zhaomin Zeng, Hongyan Liu, Qing Hu, Haiyan Yu.

**Data curation:** Hua Liao, Zhaomin Zeng, Hongyan Liu, Qing Hu.

**Formal analysis:** Hua Liao.

**Funding acquisition:** Haiyan Yu.

**Investigation:** Zhaomin Zeng, Hongyan Liu, Qing Hu.

**Supervision:** Haiyan Yu.

**Writing – original draft:** Hua Liao.

**Writing – review & editing:** Haiyan Yu.
